# Effect of motivational group interviewing-based safety education on Workers’ safety behaviors in glass manufacturing

**DOI:** 10.1186/s12889-015-2246-8

**Published:** 2015-09-19

**Authors:** Ali Navidian, Zahra Rostami, Nasrin Rozbehani

**Affiliations:** Pregnancy Health Research Center, Zahedan University of Medical Sciences, Zahedan, Iran; Arak University of medical sciences, Arak, Iran

## Abstract

**Background:**

Worker safety education using models that identify and reinforce factors affecting behavior is essential. The present study aimed to determine the effect of safety education based on motivational interviewing on awareness of, attitudes toward, and engagement in worker safety in the glass production industry in Hamedan, Iran, in 2014.

**Methods:**

This was a quasi-experimental interventional study including a total of 70 production line workers at glass production facilities in Hamedan. The workers were randomly assigned to either an intervention or a control group, with 35 workers in each group. Participants in the control group received four one-hour safety education sessions, in the form of traditional lectures. Those in the intervention group received four educational sessions based on motivational group interviewing, which were conducted in four groups of eight to ten participants each. The instruments used included a researcher-developed questionnaire with checklists addressing safety awareness, and attitude and performance, which were completed before and 12 weeks after the intervention. The data were analyzed using descriptive statistics, independent and paired *t*-tests, and chi-squared tests.

**Results:**

Having obtained the differences in scores before and after the intervention, we determined mean changes in the scores of awareness, attitude, and use of personal protective equipment among workers who underwent motivational group interviewing (3.74 ± 2.16, 1.71 ± 3.16, and 3.2 ± 1.92, respectively, *p* < 0.05). These scores were significantly greater than those of control workers who underwent traditional educational sessions (1.28 ± 1.93, 1.1 ± 3.07, and 0.2 ± 1.26, respectively).

**Conclusions:**

Our findings revealed that incorporation of motivational interviewing principles into safety education programs had the positive effect of enhancing workers’ knowledge, attitude, and, particularly, implementation of safe behaviors. The application of this advisory approach is recommended to increase workplace safety and minimize occupational hazards in the work environment.

## Background

Occupational injury and trauma are among the most important social problems for workers [1]. Despite relative improvement in the health and safety of work environments in many countries around the world, occupational injuries and their consequences for individuals and society are increasing [2]. Based on the most recent report by the Iranian Social Security Organization, the rate of work-related accidents has grown by 65 % compared with the previous year (16383 occupational injuries in 2003 versus 27031 occupational injuries in 2013), mainly owing to unsafe worker behavior and practices, and inappropriate working conditions [3].

Research demonstrates that 80–90 % of occupational accidents in industrialized countries are related to unsafe behavior and 10–20 % are related to unsafe working conditions [4]. Although the first priority is to eliminate workplace hazards through design and occupational engineering controls, human error accounts for 84–94 % of the risk factors for occupational injury in the industrial sector. One of the most common forms of error is the failure to use personal protective equipment. In fact, 34 % of occupational injuries that result in death are caused by the failure to use personal protective equipment that was available in the work environment at the time of the accident. Additionally, 13 % of workplace accidents resulting in death are caused by inappropriate use of such protective equipment [1]. Working in glass production facilities is associated with various hazards. It is necessary to observe safety behaviors, especially the use of personal protective equipment, to reduce injuries and maintain workers’ safety. Appropriate use of personal protective equipment consists of wearing safety helmets and shoes; using fireproof gloves, sleeves and aprons; using earplugs or other hearing protection in noisy environments; wearing goggles or eye protection; and wearing respirators, masks and other suitable work apparel [5].

Because worker behavior contributes to workplace accidents, educating workers about risks in the work environment and how to minimize these can help to promote worker health and safety [6]. The traditional approach to safety has focused on technical factors, such as the design, fabrication, and implementation of safety equipment, programs, and strategies. However, there has recently been greater concentration on enhancing workers’ commitment to observing safety practices. Such commitment includes following rules and regulations, wearing protective clothing and equipment, and avoiding risky behavior [7]. The financial and social costs of occupational hazards and injuries cannot be easily compared with other types of diseases. Nevertheless, the burden of workplace accidents is very high and has remained constant over recent decades [2].

Research has revealed that traditional education is ineffective under the present conditions in Iran. Behavioral change cannot be easily achieved without first recognizing and identifying the complex factors that affect behavioral changes [8, 9]. The issue of behavioral change is closely associated with factors like awareness and education, attitude, and motivation. The existence of some problems in this regard constitutes a persistent challenge to the management of safety behavior [10]. Understanding the mechanisms by which workers change their behaviors is of utmost importance. Most efforts aimed at behavioral change have failed by disregarding the psychology of change [11]. The mere presentation of information is insufficient to produce lasting behavioral change; it is also necessary to understand the factors that lead to successful behavioral change [12].

An innovative approach that has been receiving attention in recent years is motivational interviewing; increasing research shows its positive effect on behavioral change [13]. This approach is a reference-centered modality, presented in the form of instructions for reinforcing and enhancing the intrinsic motivation for behavioral change through discovery, identification, and resolving doubts and ambivalence [14]. Motivational interviewing was first described by Miller in 1938, and then further developed by Miller and Rollnick (1991) as an intervention and short-term treatment for alcoholism in cases where the patient’s lack of motivation was a barrier to treatment [15]. Owing to the positive therapeutic effects of this approach, it quickly spread to other health promotion domains in which behavioral change was an important factor and patient motivation was a common impediment to change [16]. The main goal of motivational interviewing is increasing the intrinsic motivation for change. Intrinsic motivation mainly originates from personal objectives and individual values rather than extrinsic sources like reinforcement or obligatory change. External pressure induces resistance and reduces the inclination to change or continue to change [17, 18]. In contrast, this motivational approach facilitates behavioral change through an interactive process in two stages, creating internal motivation and reinforcing the commitment to change, rather than through reasoning, providing information, advice or encouragement, or through obligation. Research shows that the motivational interviewing method is superior over traditional education and therapeutic recommendations, for a wide range of behavioral issues [19]. This modality has been successfully used for difficult or demanding interventions; motivation enhancement; treatment program completion; and to increase the effectiveness of routine practices for health behavior change [3].

Management in the industrial sector is continuously seeking better strategies for reducing occupational accidents and injuries, as well as reducing their associated costs. Worker safety education has been found to be the most appropriate method to achieve these ends. Safety education using models like motivational interviewing are crucial to identify and reinforce those factors most affecting behavior. Hence, the present study aimed to determine the effect of safety education based on motivational interviewing on enhancing awareness, attitudes, and compliance with work safety practices in glass production facilities in Hamedan, Iran. Based on this objective, we sought to find out whether the mean change scores for awareness of, and attitude and behavior toward use of personal protective equipment is significantly greater among workers who underwent motivational interviewing compared with a control group, who received traditional safety education.

## Methods

This was a quasi-experimental interventional study carried out on the basis of a before-after intervention design. The study took place from January to August 2014, and sought to determine the effect of an educational program that incorporated motivational interviewing principles on promoting the use personal protective equipment among production workers. The study population consisted of all production line workers at glass manufacturing facilities in Hamedan at the time of the study. Workers from four different shifts were randomly selected from factory employee lists. These workers were then matched for variables such as age, work experience, accident history, and work shift, and then assigned to either the intervention or control group. Based on the sample volume formula with α = 5 % and test power of 90 %, 35 subjects were randomly assigned to the intervention group and 35 to the control group; thus, the total number of study participants was 70 workers. The exclusion criteria were participation in safety education classes during the last year, a positive history of occupational injury or trauma, the presence of disease or other physical limitation, illiteracy, and absence in more than one educational session.

After explaining the objectives and procedures and obtaining informed oral consent, both groups were given a pre-test. After coordinating with factory department managers and shift schedulers, participants in the control group attended four 1-h safety education sessions that were conducted as traditional lectures. Participants in the intervention group received four educational sessions based on motivational interviewing. Sessions were conducted in four groups with eight to ten participants each. These sessions were held in the factory’s education center twice a week. The content of the classes is described in Table 1. Twelve weeks after the last education session, both groups were given a post-test. Assessment of workers’ safety performance was done by production line supervisors twice a week using a checklist. The mean scores of two assessments were used as the basis. Motivational group interviewing was carried out by the first author, and addressed variables such as lifestyle, blood pressure, obsessive-compulsive behavior, weight, and self-control with respect to eating behavior. The intervention format was based on Fields’ model [20] and further refined using books and articles related to motivational interviewing, as well as the clinical experience of the first author during his doctoral program. An occupational health specialist also assisted researchers in directing the professional discussions, for the protection and safety of participants in both the control and intervention groups.

**Table 1:** Structure and concept of educational sessions based on motivational interviewing

Session	*Educational concept*
First	***Orientation***: Introduction, group norms and process; freedom practice; practice on the influence of unsafe behavior on different life dimensions; change stages and practice on the behavioral change cycle; evaluation of commitment and confidence.
Second	***Feelings*** *:* Description of one working day: practice identifying and naming feelings; and practice recognizing the effects of unsafe behavior on family, work and social life and the feelings induced by these.
Third	***Pros and cons of behavior and change***: Assessment of short-term advantages and disadvantages of failure to use personal protective equipment; definition of values; practice recognizing and prioritizing top life values; discussing the relationship between unsafe behavior and the values governing life and developing the clear and evident discrepancy between them.
Fourth	***Perspectives and conclusion*** *:* Rewards for achievement; reinforcement of the importance of self-monitoring; recognition of situations in which it is tempting to engage in unsafe behavior and individuals’ degree of self-confidence in such situations; completion and recitation of the perspective horizon; and practice of new safe behaviors.

### Instruments

The instrument used in this study was a questionnaire developed by researchers. Some content was taken from the questionnaires of Rahmani *et al.* [9], Mohammadi zeidi *et al.* [21], and Olson *et al.* [1]. The questionnaire was further developed by including complimentary additional items, and reworded or rephrased others to suit our study objectives. The questionnaire included four parts: the first section gathered demographic information such as age, education level, work experience, and marital status. The second part included 14 items addressing topics such as knowledge about hazardous materials in the workplace, coping mechanisms against risk factors, identification of unsafe behavior and its consequences, and familiarity with personal protective equipment and safety culture. Examples of questions asked include: “What is the most harmful substance in the glass industry?” and “What is the most important factor in work accidents?” Each correct answer was worth 1 point and each wrong answer worth 0; the range of possible scores for this section of questionnaire was from 0 to 14. The third section contained six items addressing attitude. Here, attitude referred to participants’ views and beliefs about the importance of work hygiene, their thoughts, feelings and desires concerning workplace safety, and their commitment to using personal protective equipment and prioritizing safety issues. Some example items are as follows: “Sometimes it is necessary to ignore safety rules in order to speed up work and increase production.” and “I perform my duties better by ignoring some instructions.” A Likert five-point scale was used for this part of the questionnaire. In positive items (1, 4 & 5), five points were given for “I agree completely” and 1 point was given for “I disagree completely”. In negative items (2, 3 & 6) the points were given in reverse. The score range in this section of the questionnaire was from 6 to 30. Those scoring higher demonstrated a more suitable attitude towards the use of personal protective equipment. The fourth section included a checklist with seven items for assessing behavioral performance. The behavioral checklist included items such as the use of suitable gloves, helmets, respirators, safety shoes, hearing protection devices, work clothing, and goggles or protective face shields. Example items included, “Do you wear a helmet?” and “Do you wear suitable work clothing?” Answers for this checklist were “Yes”, “No”, and “Sometimes”, with respective point values of 2, 0, and 1; scores for this section ranged from 0 to 14. Those with higher scores for this part of the questionnaire were considered to have a better attitude towards the use of personal protective equipment.

A panel of 10 experts in health education, and occupational health and safety analyzed the validity of the questionnaire’s contents using qualitative and quantitative methods. For the qualitative method, the experts were asked to assess the instrument based on correct grammar usage, choice of appropriate diction, appropriate sequencing of items, and proper weighting of items, and also to provide feedback. For the quantitative assessment, the Content Validity Ratio (CVR) and Content Validity Index (CVI) were determined. To determine CVR, experts were asked to give their opinion regarding the need for (or lack thereof) each item. CVR values of 0.71, 0.77, and 0.86 were considered acceptable for awareness, attitude, and performance, respectively. To assess CVI, experts reviewed each item for relevance, clarity, and simplicity. CVI values of 0.81, 0.88, and 0.92 were considered acceptable for awareness, attitude, and performance, respectively.

In this study, the reliability coefficient of the questionnaire was measured for 30 workers who were similar to our study population in terms of demographic features. Cronbach’s alpha was estimated to be 0.82 and 0.76 for attitude and awareness items, respectively. To calculate the reliability coefficient of the performance assessment checklist, the inter-rater correlation coefficient was estimated to be 0.88, which was acceptable.

### Data analysis

The data were analyzed using IBM SPSS (Statistical Package for the Social Sciences), Version 21.0 (IBM Corp., Armonk, NY, USA). All comparisons were two-tailed and *p*-values <0.05 were considered significant. Descriptive statistics for the various variables, such as percentages, means, and standard deviations, were used to describe the sample. To compare variables between the control and intervention groups, an independent *t*-test (for quantitative variables) and chi-squared test (for qualitative variables) were used.

### Ethical considerations

The current study was approved in 2014 by the Research Ethical Committee of Arak University of Medical Sciences in Iran. Ethical considerations of the study included the methods and tools used, aim of study, duration of the intervention, obtaining oral informed consent, confidentiality of information, and participants’ right to withdraw from the study at will.

## Results

The minimum and maximum age of participants was 19 and 54 years, respectively. Mean age of the control group was 33.28 ± 7.32 years and that of the intervention group was 32.68 ± 5.56 years. The minimum and maximum years of work experience was 2 and 26, respectively; the mean for the intervention group was 5.07 ± 7.08 years and 5.38 ± 7.68 years for the control group. The independent *t*-test showed that there was no statistically significant difference between the two groups (*p* < 0.05) with respect to age and work experience. Regarding education level, 57.1 % of the intervention group and 54.3 % of the control group had a high school diploma or higher degree. Additionally, 94.2 % of the intervention group and 97.1 % of the control group were employed under a contract. The results of the chi-squared test revealed that the two groups were not significantly different with respect to education level and type of employment (*p* < 0.05).

The results of the independent *t*-test demonstrated that there was no significant difference between workers in the two groups with respect to mean scores for awareness, attitude, and compliance with use of personal protective equipment before the motivational interviewing intervention (*p* < 0.05). According to Table 2, although there was a significant increase in awareness in both groups after the intervention compared with before; But the changing of mean of awareness core in intervention group (3/74 ± 2/16) was more than the changing of mean of awareness score in control group (1/28 ± 1/93), significantly (*p* = 0.01).

**Table 2:** Comparison of mean and standard deviation (SD) of the score for “Awareness about the use of personal protective equipment” before and after motivational group interviewing, for the intervention and control groups

Time	Before	After	Changes	Paired *T*-test
Group	Mean & SD	Mean & SD	Mean & SD	
Experimental	8.42 ± 1.71	12.17 ± 2.03	3.74 ± 2.16	0.001
Control	8.74 ± 2.09	10.02 ± 1.91	1.28 ± 1.93	0.04
Independent *T*-test	0.49	0.01	0.01	

Table 3 further demonstrates that although there was a significant increase in the attitude scores for both groups after motivational group interviewing compared with before the intervention, the mean workers’ attitude score in the intervention group was 28.40 ± 1.95, which showed a statistically significant difference compared with the control group score of 27.32 ± 4.32 (*p* = 0.02).

**Table 3:** Comparison of mean and standard deviation (SD) of the score for “Attitude towards the use of personal protective equipment” before and after motivational group interviewing, for the intervention and control groups

Time	Before	After	Changes	Paired *T*-test
Group	Mean & SD	Mean & SD	Mean & SD	
Experimental	26.68 ± 4.10	28.40 ± 1.95	1.71 ± 3.16	0.003
Control	26.20 ± 3.10	27.32 ± 2.3	1.1 ± 3.07	0.07
Independent *T*-test	0.72	0.05	0.02	

Moreover, as Table 4 reveals, the mean score for engaging in the use of personal protective equipment among workers in the intervention group was 11.71 ± 2.13, which was significantly lower than that of workers in the control group (8.22 ± 2.24, *p =* 0.001).

**Table 4:** Comparison of mean and standard deviation (SD) of the score for “engaging in the use of personal protective equipment” before and after motivational group interviewing, for the intervention and control groups

Time	Before	After	Changes	Paired *T*-test
Group	Mean & SD	Mean & SD	Mean & SD	
Experimental	8.56 ± 2.18	11.71 ± 2,13	3.2 ± 1.92	0.001
Control	8.07 ± 2.16	8.22 ± 2.24	0.2 ± 1.26	0.23
Independent *T*-test	0.15	0.001	0.001	

Having calculated the differences between participants’ scores before and after the intervention, our general findings showed that the mean change and increase in scores for awareness, attitude, and use of personal protective equipment were significantly greater among workers in the intervention group after motivational group interviewing compared with those in the control group.

## Discussion

The findings of this study indicated that although there was increased awareness and attitudes about safety among participants in both the intervention and control groups, the rates of awareness and positive changes in attitude were significantly greater in the intervention group compared with the control group, who received traditional training. This highlights the superiority of motivational interviewing over traditional education methods with respect to enhancing awareness and attitudes about workplace safety.

Our findings indicate that safety instruction leads to increased awareness of safety issues and helps to create a positive attitude about safety among workers. Many studies have demonstrated that theory-based education results in greater awareness and more positive attitudes about safe behaviors compared with traditional instruction [22, 23]. Although it is expected that traditional instructional methods would lead to increased awareness and safety knowledge, as is the case with motivational interviewing, our study showed that there was no significant change in workers’ attitudes after compared with before the intervention in control group. Thus, traditional instruction is considered insufficient for affecting behavioral change. When compared with traditional education, educational (instructional) interventions based on motivational interviewing have a greater effect on increasing safety awareness and attitudes, which can be attributed to the therapeutic elements of motivational interviewing. Arkowitz [24] believed that motivational interviewing, if integrated into other common educational and therapeutic methods, can produce a synergistic effect, increasing the effectiveness of other methods by increasing therapeutic commitment [24].

With respect to our results in terms of safety behavior, our findings revealed that motivational group interviewing increased the level of safety behavior performance and the use of personal protective equipment among workers post-intervention compared with pre-intervention. However, workers in the control group showed no increase in safety behaviors, indicating the considerable effectiveness of motivational interviewing and the ineffectiveness of traditional education in enhancing safety behaviors.

There have been differing results with respect to the effect of traditional education on promoting safe behaviors. A study by Amidi Mazaheri *et al.* [25] showed that instruction using the lecturing method increased the level of safe behaviors, which is inconsistent with the findings of the present study. Other studies, like Tajvar *et al.* [26], found that education based on traditional methods slightly decreased unsafe behaviors, from 78.2 to 67.9 % after instruction. Conversely, in line with our findings regarding the inability of traditional education methods to considerably promote safety behavior, Taghdisi *et al.* [27] asserted that although conventional training can somewhat increase awareness levels, such instruction does not always lead to a change in behavior.

Safety instruction based on health education models and behavioral change theories are more effective in changing behavior [28, 29]. The findings of Mohammadi-Zeidi *et al.* (2013) showed that theory-based educational intervention leads to improved dimensions of the atmosphere of safety in work environments [21]. Al-Hemood and Al-Asfoor [30], and Joshua and Geller [31] revealed that the average workers’ safe behaviors increased after intervention by 26 % and 9 %, respectively, which is consistent with our finding that motivational interviewing promoted the mean safe behavior performance score by 36.8 %.

Motivational interviewing offers a greater possibility for behavioral change because its processes (collaboration, evocation, autonomy), principles (expressing empathy, developing discrepancy, rolling with resistance, and supporting self-efficacy) and skills (open-ended questions, listening reflectively, summarizing, affirming, and eliciting change talk) help individuals to resolve their ambivalence and enhance their motivation for change. In this way, motivational interviewing is effective in promoting behavioral change [16, 24]. Meta-analyses of several randomized clinical trials investigating the effectiveness of motivational interviewing have demonstrated that individuals who underwent motivational interviewing showed significantly greater changes in their behavior compared with those who received no such education; the magnitude of this change reached 0.7 in some of these studies [32, 33]. The effectiveness of motivational interviewing has been proved in studies dealing with reducing high risk sexual behavior [34], encouraging workers to return to work after illness [35], promotion of physical activity and quality of life [36], self-efficacy of eating behaviors [17], enhancement of breast-feeding behavior [37], changing hygiene habits among elderly adults [38], increasing the consumption of fruits and vegetables [39], medication compliance [40], and many other action-centered behaviors. Because motivational interviewing removes the pressure for change, it may better prepare individuals to more effectively confront barriers to change [24].

Overall, our findings demonstrated that traditional education only increases levels of awareness about safe behaviors and does not significantly influence safe behavior attitudes and performance. However, safety education that is based on motivational interviewing, specifically in a group format, affects not only safety knowledge levels but also enhances the attitudes about and performance of behaviors related to work safety.

### Limitations of the study

The present study had some limitations, the most important of which is the following: our results may have been improved by including a third participant group (as “no intervention group”). Focusing on only male participants and absence of long-term follow-up of the effects of the intervention are among the limitations of the current research. Although the results of the current study can be used to promote increasing personal protective equipment use to prevent and reduce workplace accidents and injuries, its application to industry-based studies is limited.

## Conclusion

Overall, we conclude that worker education and instruction, based on a proper design and innovative theories of behavioral change, such as motivational interviewing, can have a noticeable effect on enhancing the safety level of workers and, ultimately, the safety of the community in general. Consequently, it is recommended that this type of education be applied to other studies and industrial work environments, to increase workplace safety and decrease occupational hazards and injuries, as well as their associated costs, for workers in industrial professions.

### Availability of data and materials

Not applicable.
